# The Impact of Down Syndrome on Perioperative Anesthetic Management and Outcomes in Infants Undergoing Isolated Ventricular Septal Defect Closure

**DOI:** 10.3390/diagnostics15151839

**Published:** 2025-07-22

**Authors:** Serife Ozalp, Funda Gumus Ozcan

**Affiliations:** Department of Anesthesiology and Reanimation, Istanbul Health Sciences University Basaksehir Cam and Sakura Hospital, Istanbul 34480, Turkey; fgumus@hotmail.com

**Keywords:** down syndrome, ventricular septal defect, congenital heart disease, pediatric cardiac anesthesia, postoperative outcomes

## Abstract

**Background:** Down syndrome (DS) is associated with unique anatomical and physiological characteristics that complicate the perioperative management of infants undergoing cardiac surgery. While ventricular septal defect (VSD) repair is commonly performed in this population, detailed data comparing perioperative outcomes in DS versus non-syndromic infants remain limited. **Methods:** This retrospective matched study analysed 100 infants (50 with DS and 50 without DS) who underwent isolated VSD closure between January 2021 and January 2025. Patients were matched by age and surgical date. Intraoperative anesthetic management, complications, postoperative outcomes, and mortality were compared between groups. **Results:** DS patients had lower age, weight, and height at surgery. They required significantly smaller endotracheal tube sizes, more intubation and vascular access attempts. The DS group had significantly lower rates of extubation in the operating room and experienced longer durations of mechanical ventilation and ICU stay. However, no significant differences were observed in total hospital stay or mortality between groups. **Conclusions:** Although DS infants present with increased anesthetic complexity and respiratory challenges, they do not exhibit higher surgical mortality following isolated VSD closure. Tailored perioperative strategies may improve respiratory outcomes in this high-risk group.

## 1. Introduction

Down syndrome (trisomy 21) is the most common chromosomal abnormality, affecting approximately 1 in 700 live births [1,2]. Cardiovascular disease is a leading cause of morbidity and mortality in individuals with Down syndrome. Among these, congenital heart disease (CHD) is the most frequent cardiovascular condition, occurring in up to 40–60% of patients with Down syndrome and contributing significantly to adverse clinical outcomes [3,4,5]. The most common cardiac defects include complete atrioventricular septal defect, atrial and ventricular septal defects, and tetralogy of Fallot. Among these, ventricular septal defect (VSD) is the most frequently encountered [6]. The vast majority of Down syndrome (DS) patients require cardiac surgery due to CHD. This syndrome is associated with multiple comorbidities and is considered a significant risk factor for early development of pulmonary vascular disease and pulmonary hypertension, especially in individuals with CHD [2].

Ventricular septal defect (VSD) is the most common CHD in the infant age group and often requires surgical repair at an early age. Infants with Down syndrome present unique anatomical and physiological characteristics that necessitate special attention in anesthetic and surgical management. Differences in airway anatomy, difficulties in vascular access, predisposition to pulmonary hypertension, and variable responses to anesthetic agents make the perioperative process more complex in this population. Previous studies evaluating outcomes of congenital cardiac surgery in patients with Down syndrome have presented conflicting results. Some have reported increased mortality, longer hospital stays, and prolonged ventilation, while others have shown comparable or even better outcomes in DS patients [7,8,9]. Although some studies have examined surgical outcomes in children with Down syndrome, there is limited literature focusing specifically on intraoperative anesthetic management and early postoperative outcomes in infants undergoing isolated VSD closure.

In this retrospective study, we compared intraoperative anesthetic techniques, complication rates, postoperative mechanical ventilation duration, morbidity, and mortality between infants with and without Down syndrome undergoing isolated VSD closure at our institution. Age- and weight-matched groups were used to isolate the effect of Down syndrome as an independent variable.

## 2. Method

This retrospective study included infants aged 0–12 months who underwent isolated ventricular septal defect closure at a tertiary cardiac surgery center between January 2021 and January 2025. Patients were identified through the hospital record system and electronic archives.

Inclusion criteria are as follows:

- Age ≤ 12 months at the time of surgery;
- Isolated VSD closure performed.
- Exclusion criteria are as follows:
- Patients intubated preoperatively;
- Diagnosis of genetic syndromes other than Down syndrome;
- Additional cardiac anomalies requiring concomitant surgical intervention;
- Patients older than 12 months.

**Figure 1:**
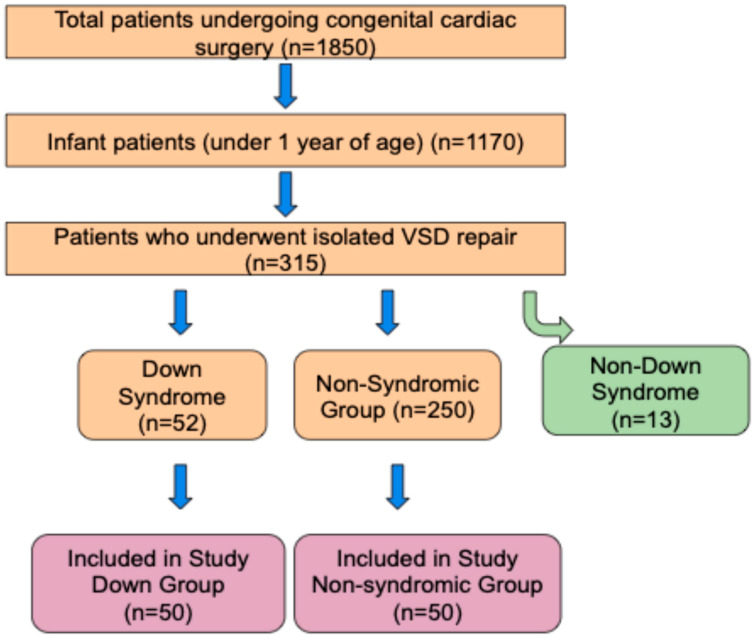
Consort flow diagram.

According to these criteria, 52 DS and 250 non-syndromic infant patients were identified. Fifty DS patients were matched with 50 non-DS patients based on similar surgical dates (±30 days) to form two groups. To minimize selection bias and ensure comparability between groups, 50 non-DS patients were selected from the 250 eligible non-syndromic infants based on a one-to-one manual matching process. (Figure 1) Matching was primarily performed according to surgical date (±30 days). Whenever possible, cases operated on by the same surgeon and managed by the same anesthesiologist were preferentially selected to reduce variability due to operator-dependent factors. The matching process was conducted by two independent researchers blinded to postoperative outcomes. When multiple non-DS candidates met the criteria for a single DS case, the patient whose surgery date and age most closely approximated that of the DS patient was selected. This approach ensured that potential confounders, such as surgical technique evolution or seasonal variations in care, were minimized. Propensity score matching was not utilised due to the relatively small number of DS cases available.

Upon arrival in the operating room, standard monitoring (ECG, non-invasive blood pressure, and pulse oximetry) was initiated before anesthetic induction. The induction regimen included:

- Midazolam (0.1 mg/kg);
- Fentanyl (1 µg/kg);
- Ketamine (1 mg/kg);
- Rocuronium bromide (0.6 mg/kg).

Additionally, cefazolin (30 mg/kg IV) and methylprednisolone (3 mg/kg IV) were administered as standard prophylactic medications.

For purposes of this study, a vascular access attempt was defined as the number of distinct needle insertions through the skin during central venous catheterization or invasive arterial cannulation. All vascular access procedures were performed under real-time ultrasound guidance by experienced anesthesiologists. The number of attempts was recorded until successful placement and confirmation of a functioning catheter or arterial line. In addition, any access-related complications, such as hematoma, pneumothorax or arterial puncture, were documented.

Data collected included demographic characteristics, anesthetic techniques, number of vascular access attempts, drugs administered, hemodynamic parameters, intraoperative durations, and complications. Postoperative outcomes, including mechanical ventilation duration, ICU and hospital stay length, and mortality, were also analyzed.

This study was approved by the University of Health Sciences Türkiye, Basaksehir Cam and Sakura City Hospital Local Ethics Committee (Approval Number: KAEK/07.05.2025.141).

### Statistical Analysis

Statistical analyses were performed using R version 4.4.2 (R Foundation for Statistical Computing, Vienna, Austria), incorporating various packages for data management, visualization, and reporting. The R6 package (version 2.5.1, RStudio, Boston, MA, USA) was used to build reusable object-oriented structures, perform rstatix-facilitated statistical testing (version 0.7.2, Alboukadel Kassambara, France), and generate publication-ready summary tables using the flextable package (version 0.9.5, David Gohel, France).

Inferential statistics were used to assess relationships and differences between groups. Data normality was evaluated using the Shapiro–Wilk test. For normally distributed numerical data, *t*-tests or ANOVA were used. For non-normal distributions, Wilcoxon rank-sum or Kruskal–Wallis tests were applied. Categorical data were analyzed using Chi-square tests or Fisher’s exact test, depending on sample size. A *p*-value < 0.05 was considered statistically significant.

## 3. Results

This retrospective matched study included 100 infants who underwent isolated VSD closure between January 2021 and January 2025. The cohort consisted of 50 DS and 50 non-DS patients. Mean age was 5.2 ± 2.8 months, mean height was 61.2 ± 6.1 cm, and mean weight was 5.3 ± 1.6 kg. Gender distribution was 58% male and 42% female. Patients in the DS group had significantly lower mean age, weight, and height compared to the non-DS group (*p* = 0.017, *p* = 0.009, and *p* = 0.013, respectively). No significant difference was observed in gender distribution (*p* = 0.543) (Table 1).

The DS group required significantly smaller endotracheal tube sizes (*p* < 0.001), and more attempts at intubation (*p* = 0.031), central venous catheterization (*p* = 0.041), and arterial line placement (*p* < 0.001). Although the need for cutdown arterial access was higher in the DS group, it did not reach statistical significance (*p* = 0.056). No significant differences were found in the use of packed red blood cells, platelets, cryoprecipitate, or fibrinogen between the groups (all *p* > 0.05).

Mean operative time was 216 ± 50 min, anesthesia time 305 ± 49 min, cardiopulmonary bypass (CPB) time 85.5 ± 28.8 min, and cross-clamp time 67.6 ± 27.1 min. There were no significant differences between groups in any intraoperative duration (all *p* > 0.05) (Table 2).

Extubation in the operating room was significantly more common in the non-DS group (*p* < 0.001). The mean postoperative mechanical ventilation time was 14.5 ± 19.2 h, ICU stay 4.4 ± 3.5 days, and hospital stay 9.9 ± 4.1 days. The DS group had significantly longer ventilation times (median: 17 vs. 0 h, *p* < 0.001) and ICU stays (median: 5 vs. 3 days, *p* = 0.001). However, no significant difference was observed in total hospital stay (*p* = 0.301) or mortality rate (2% in both groups, *p* > 0.999) (Table 3).

## 4. Discussion

In this retrospective matched study, intraoperative anesthetic management and early postoperative outcomes were compared between infants with Down syndrome (DS) and those without (non-DS) undergoing isolated ventricular septal defect (VSD) closure. Although both groups underwent similar surgical protocols and were operated on in a controlled institutional setting, our findings revealed distinct differences in airway management, vascular access, and postoperative respiratory support needs, underscoring the nuanced challenges posed by the DS population.

Patients in the DS group were significantly younger, shorter, and lighter than their non-DS counterparts at the time of surgery. This is consistent with the well-established growth retardation observed in infants with trisomy 21 [10,11,12] and has important implications for perioperative dosing, equipment selection, and temperature regulation.

Airway management was more challenging in the DS group, characterized by smaller endotracheal tube (ETT) sizes and a higher number of intubation attempts. These findings are consistent with previous studies indicating that anatomical differences in the upper airway and neck structures in DS patients contribute to difficult intubation [13,14,15,16,17,18]. Common anatomical abnormalities—including macroglossia, midface hypoplasia, subglottic stenosis, tracheomalacia, and laryngomalacia demand a tailored approach. Standard age-based formulas for ETT sizing may be unreliable; thus, preoperative preparation should involve a wide range of tube sizes, including uncuffed tubes in smaller calibers. In addition to anatomical airway challenges, cervical spine instability—particularly atlantoaxial instability—is a well-recognized concern in infants with Down syndrome. Therefore, minimizing neck extension during laryngoscopy is critical. The use of videolaryngoscopy is strongly recommended in this population to reduce intubation attempts, assist in accurate endotracheal tube sizing, and limit cervical spine manipulation, thereby reducing the risk of iatrogenic injury. Failure to anticipate these difficulties may lead to airway trauma, prolonged intubation, and a higher risk of respiratory complications.

Our study also revealed significant challenges in achieving successful vascular access in DS infants. Both central venous and arterial catheterizations required more attempts in the DS group, likely due to the presence of anatomical variations, shorter necks, low muscle tone and excess adipose tissue, which obscure vascular landmarks [19]. In fact, based on our institutional experience, we now recommend the routine use of ultrasound guidance for all vascular access procedures—including peripheral venous, central venous, and arterial catheterizations—in infants with Down syndrome. This approach aims to minimize the number of attempts, avoid delays, and reduce complications, such as hematoma formation or inadvertent arterial puncture. Even in routine cases, this proactive strategy may alleviate procedural stress and improve the overall efficiency of intraoperative workflow.

Despite the technical challenges encountered during induction and line placement, no significant differences were found between the two groups in terms of intraoperative durations, including anesthesia time, surgical time, cardiopulmonary bypass (CPB) time, and aortic cross-clamp time. Similarly, transfusion requirements did not differ significantly. This may reflect the uniformity in surgical technique and anesthetic protocols at our institution, as well as the benefits of having a consistent team of experienced pediatric cardiac anesthesiologists and surgeons. Similar findings regarding bleeding and transfusion requirements in DS and non-DS patients have been reported [20]. Conversely, some studies reported significant differences in intraoperative durations and transfusion volumes between DS and non-DS groups undergoing VSD repair [21]. In our cohort, no patients in either group required temporary or permanent pacemaker insertion following VSD repair. Although some studies have reported an increased risk of postoperative conduction abnormalities in patients with Down syndrome, our findings did not reflect this trend, possibly due to surgical technique consistency and the relative simplicity of isolated VSD closure procedures.

In our study, the rate of on-table extubation was significantly lower in the DS group. In our clinical practice, the decision to extubate on the operating table is made through a multidisciplinary consensus involving the pediatric cardiologist, pediatric cardiac surgeon, and anesthesiologist. This is typically performed after epicardial echocardiographic assessment at the end of the surgical procedure. Infants with signs or a preoperative diagnosis of pulmonary hypertension are generally not considered for immediate extubation in the operating room. Although our retrospective data did not consistently include quantitative pulmonary artery pressures, our institutional experience suggests that the presence of pulmonary hypertension—more common in the DS group—was the primary factor influencing delayed extubation. This limitation has been acknowledged, and we highlight the need for future prospective studies with standardized preoperative hemodynamic assessments.

Furthermore, these patients experienced longer durations of mechanical ventilation and intensive care unit (ICU) stay. These findings are in line with previous reports indicating increased risks of prolonged mechanical ventilation and extubation failure in infants with DS after cardiac surgery [5,22]. These findings likely stem from both anatomical and functional vulnerabilities, including a propensity for airway obstruction and pulmonary hypertension, all of which are more prevalent in DS infants. Given their increased risk of extubation failure and respiratory complications, it is advisable to individualize weaning protocols and consider non-invasive ventilation support early in the postoperative course. In addition, the use of sedation-sparing techniques and multimodal analgesia can aid in respiratory stability and facilitate smoother recovery. However, other studies have not demonstrated an association between DS and prolonged ventilation or ICU stay following cardiac procedures [23,24].

From an anesthetic perspective, our findings highlight the importance of a comprehensive, multidisciplinary perioperative plan tailored specifically to the DS population. Preoperative assessments should extend beyond cardiac imaging to include detailed airway evaluation and vascular access planning. Early communication with the ICU team and respiratory therapists is essential for establishing extubation readiness criteria and anticipating postoperative support needs. Employing enhanced recovery pathways, adjusted for the unique physiology of DS infants, may further standardize and improve outcomes.

Despite the airway and vascular access challenges, overall surgical outcomes in DS infants undergoing isolated VSD repair were favorable. Notably, there was no observed increase in mortality among DS patients, which is consistent with other reports showing similar mortality rates between DS and non-DS groups following congenital heart surgery [25,26,27,28]. These results suggest that with experienced teams and appropriate adaptations in anesthetic and surgical management, DS infants can achieve outcomes on par with their non-syndromic peers. Our findings contribute to the growing body of literature advocating for individualized, risk-adjusted care protocols in this vulnerable yet resilient population.

Limitations: This study is limited by its retrospective and single-center design, which may affect the generalizability of findings to other institutions with different care models or patient populations. Although patients were carefully matched for surgical timing and demographic variables, unmeasured confounders—such as variations in the genetic expression of trisomy 21 or undocumented comorbidities—may have influenced the outcomes. Furthermore, we were unable to perform a meaningful comparison of Down syndrome–associated conditions (e.g., immune dysfunction, central nervous system abnormalities, gastrointestinal complications) due to the limited sample size and low event rates. Larger, multicenter cohorts are needed to explore the potential impact of these conditions on perioperative outcomes more comprehensively.

In conclusion, this study underscores the anesthetic and perioperative challenges associated with managing infants with Down syndrome undergoing isolated VSD closure. These challenges—ranging from airway management and vascular access to extubation planning—necessitate a meticulous, syndrome-specific approach. Anticipating smaller ETT requirements, utilizing ultrasound guidance for invasive procedures, preparing for postoperative respiratory support, and ensuring consistency in care delivery are essential strategies for optimizing outcomes. As advances in congenital cardiac surgery continue to improve survival, attention must shift toward refining anesthetic practices that support both short-term recovery and long-term developmental health. Prospective multicenter studies are warranted to develop and validate standardized anesthetic guidelines tailored to the DS pediatric cardiac population.

## Figures and Tables

**Table 1:** Demographic variables.

	Overall (*n* = 100)	Non-Syndromic (NS) (*n* = 50)	Down Syndrome (D) (*n* = 50)	*p*
Age (months)				0.017
*Mean*	5.2 ± 2.82	5.87 ± 2.99	4.53 ± 2.48	
*Median*	4.5 (3–6.53)	5.25 (3.62–7.38)	3.95 (3–5.75)	
Sex				0.543
*Male*	58 (58.00%)	27 (54.00%)	31 (62.00%)	
*Female*	42 (42.00%)	23 (46.00%)	19 (38.00%)	
Height (cm)				0.013
*Mean*	61.23 ± 6.09	62.72 ± 6.84	59.71 ± 4.84	
*Median*	61 (56–65)	63 (57–68)	60 (56–63)	
Weight (kg)				0.009
*Mean*	5.3 ± 1.57	5.74 ± 1.74	4.87 ± 1.25	
*Median*	5 (4.07–6.21)	5.9 (4.4–6.88)	4.55 (3.92–5.75)	

**Table 2:** Intraoperative variables.

	Overall (*n* = 100)	Non-Syndromic (NS) (*n* = 50)	Down Syndrome (D) (*n* = 50)	*p*
Endotracheal Tube Size				<0.001
*3*	1 (1.00%)	0 (0.00%)	1 (2.00%)	
*3.5*	19 (19.00%)	4 (8.00%)	15 (30.00%)	
*4*	38 (38.00%)	15 (30.00%)	23 (46.00%)	
*4.5*	40 (40.00%)	30 (60.00%)	10 (20.00%)	
*5*	2 (2.00%)	1 (2.00%)	1 (2.00%)	
Number of Intubation Attempts				0.031
*1*	91 (91.00%)	49 (98.00%)	42 (84.00%)	
*2*	8 (8.00%)	1 (2.00%)	7 (14.00%)	
*3*	1 (1.00%)	0 (0.00%)	1 (2.00%)	
Number of Central Line Attempts				0.041
*1*	4 (4.00%)	1 (2.00%)	3 (6.00%)	
*2*	81 (81.00%)	46 (92.00%)	35 (70.00%)	
*3*	8 (8.00%)	2 (4.00%)	6 (12.00%)	
*>3*	7 (7.00%)	1 (2.00%)	6 (12.00%)	
Number of Arterial Line Attempts				<0.001
*1*	9 (9.00%)	1 (2.00%)	8 (16.00%)	
*2*	76 (76.00%)	48 (96.00%)	28 (56.00%)	
*3*	13 (13.00%)	1 (2.00%)	12 (24.00%)	
*>3*	2 (2.00%)	0 (0.00%)	2 (4.00%)	
Need for Arterial Cutdown				0.056
*No*	95 (95.00%)	50 (100.00%)	45 (90.00%)	
*Yes*	5 (5.00%)	0 (0.00%)	5 (10.00%)	
Total Packed Red Blood Cells Used (ml)				0.750
*Mean*	35.1 ± 26.65	35 ± 31.25	35.2 ± 21.4	
*Median*	30 (20–50)	30 (0–50)	30 (30–50)	
Need for Platelets				0.617
*No*	96 (96.00%)	49 (98.00%)	47 (94.00%)	
*Yes*	4 (4.00%)	1 (2.00%)	3 (6.00%)	
Need for Cryoprecipitate				0.254
*No*	74 (74.00%)	40 (80.00%)	34 (68.00%)	
*Yes*	26 (26.00%)	10 (20.00%)	16 (32.00%)	
Need for Fibrinogen				0.413
*No*	84 (84.00%)	40 (80.00%)	44 (88.00%)	
*Yes*	16 (16.00%)	10 (20.00%)	6 (12.00%)	
Operation Time (min)				0.975
*Mean*	216.05 ± 50.19	214.54 ± 40.39	217.59 ± 58.92	
*Median*	215 (182.5–240)	212.5 (181.25–240)	220 (185–240)	
Anesthesia Time (min)				0.992
*Mean*	305.45 ± 49.73	302.9 ± 42.8	308.06 ± 56.27	
*Median*	300 (275–327.5)	300 (276.25–323.75)	300 (275–330)	
Cardiopulmonary Bypass Time (min)				0.761
*Mean*	85.51 ± 28.82	83.02 ± 22.01	88.04 ± 34.47	
*Median*	83 (69–98)	81 (67.25–98)	87 (70–98)	
Cross-Clamp Time (min)				0.986
*Mean*	67.59 ± 27.07	65.66 ± 19.51	69.55 ± 33.17	
*Median*	66 (52.5–79)	67 (54.25–77)	65 (51–79)	

**Table 3:** Postoperative variables.

	Overall (*n* = 100)	Non-Syndromic (NS) (*n* = 50)	Down Syndrome (D) (*n* = 50)	*p*
Initial Postoperative ABG Lactate				0.790
*Mean*	3.01 ± 9.04	2.19 ± 1.09	3.83 ± 12.75	
*Median*	2 (1.4–2.4)	2 (1.5–2.3)	2 (1.4–2.68)	
Extubation in OR				<0.001
*No*	55 (55.00%)	16 (32.00%)	39 (78.00%)	
*Yes*	45 (45.00%)	34 (68.00%)	11 (22.00%)	
Mechanical Ventilation Duration (hours)				<0.001
*Mean*	14.55 ± 19.24	9.18 ± 16.38	19.92 ± 20.51	
*Median*	6 (0–24)	0 (0–14.5)	17 (5–24)	
Vasoactive–Inotropic Score				0.455
*Mean*	1.09 ± 0.32	1.06 ± 0.24	1.12 ± 0.39	
*Median*	1 (1–1)	1 (1–1)	1 (1–1)	
ICU Length of Stay (days)				0.001
*Mean*	4.43 ± 3.53	4.12 ± 4.53	4.74 ± 2.11	
*Median*	4 (2–6)	3 (2–4)	5 (3–6)	
Hospital Length of Stay (days)				0.301
*Mean*	9.91 ± 4.06	10.1 ± 4.99	9.72 ± 2.88	
*Median*	9 (8–11)	8 (7–10)	9 (8–11.75)	
Mortality				>0.999
Survived	98 (98.00%)	49 (98.00%)	49 (98.00%)	
Ex	2 (2.00%)	1 (2.00%)	1 (2.00%)	

## Data Availability

All data used in this study can be obtained from the corresponding author upon request.
